# Within- and Between-Session Prefrontal Cortex Response to Virtual Reality Exposure Therapy for Acrophobia

**DOI:** 10.3389/fnhum.2018.00362

**Published:** 2018-11-01

**Authors:** Aleksandra Landowska, David Roberts, Peter Eachus, Alan Barrett

**Affiliations:** ^1^Department of Psychology, School of Health Sciences, University of Salford, Salford, United Kingdom; ^2^Military Veterans’ Service, Pennine Care NHS Foundation Trust, Ashton-under-Lyne, United Kingdom

**Keywords:** VRET, prefrontal cortex, fNIRS, phobia, virtual reality, anxiety

## Abstract

Exposure Therapy (ET) has demonstrated its efficacy in the treatment of phobias, anxiety and Post-traumatic Stress Disorder (PTSD), however, it suffers a high drop-out rate because of too low or too high patient engagement in treatment. Virtual Reality Exposure Therapy (VRET) is comparably effective regarding symptom reduction and offers an alternative tool to facilitate engagement for avoidant participants. Neuroimaging studies have demonstrated that both ET and VRET normalize brain activity within a fear circuit. However, previous studies have employed brain imaging technology which restricts people’s movement and hides their body, surroundings and therapist from view. This is at odds with the way engagement is typically controlled. We used a novel combination of neural imaging and VR technology—Functional Near-Infrared Spectroscopy (fNIRS) and Immersive Projection Technology (IPT), to avoid these limitations. Although there are a few studies that have investigated the effect of VRET on a brain function after the treatment, the present study utilized technologies which promote ecological validity to measure brain changes after VRET treatment. Furthermore, there are no studies that have measured brain activity within VRET session. In this study brain activity within the prefrontal cortex (PFC) was measured during three consecutive exposure sessions. *N* = 13 acrophobic volunteers were asked to walk on a virtual plank with a 6 m drop below. Changes in oxygenated (HbO) hemoglobin concentrations in the PFC were measured in three blocks using fNIRS. Consistent with previous functional magnetic resonance imaging (fMRI) studies, the analysis showed decreased activity in the DLPFC and MPFC during first exposure. The activity increased toward normal across three sessions. The study demonstrates potential efficacy of a method for measuring within-session neural response to virtual stimuli that could be replicated within clinics and research institutes, with equipment better suited to an ET session and at fraction of the cost, when compared to fMRI. This has application in widening access to, and increasing ecological validity of, immersive neuroimaging across understanding, diagnosis, assessment and treatment of, a range of mental disorders such as phobia, anxiety and PTSD or addictions.

## Introduction

Specific phobia is a psychiatric disorder characterized as a persistent fear that is either unreasonable or excessive, caused by the presence or anticipation of a specific object or situation (DSM—V American Psychiatric Association, 2013). Previous clinical trials demonstrated that *in vivo* Exposure Therapy (ET) appears to be a more effective treatment for specific phobias compared to imaginal ET and wait-list control group or placebo (Choy et al., 2007). However traditional *in vivo* and imaginal ET have many disadvantages. *In vivo* ET has limited control of the exposure situation, could be expensive and take a lot of time (Riva, 2005). Additionally, it puts patients in a potentially distressing situation, where they need to face the real threat or embarrassment related to some public aspects of *in vivo* treatment (Rizzo et al., 2007). On the other hand, during imaginal ET some patients are reluctant, or find it difficult to mentally visualize the fear-inducing stimuli, or are hesitant to verbally engage with the therapist in imaginal ET (Rothbaum and Hodges, 1999; Garcia-Palacios et al., 2002; Rothbaum et al., 2006; Rizzo et al., 2013). Furthermore, in anxiety disorders or Post-traumatic Stress Disorder (PTSD) some patients are unwilling or unable to sufficiently engage emotionally during the therapy and visualize or recall the anxiogenic stimuli or traumatic event (Rothbaum and Hodges, 1999; Rizzo et al., 2013). Previous studies on ET indicated that excessive, or the lack of emotional engagement during a therapy session could be a predictor for negative treatment results (Jaycox et al., 1998; Rothbaum and Schwartz, 2002).

Virtual Reality Exposure Therapy (VRET) employs VR technology in order to mediate ET controlled by a therapist (Rothbaum et al., 1996; Rothbaum and Hodges, 1999). It elicits fear-related responses in an objectively safe context which resembles real-life situations and enables a patient to interact with three-dimensional virtual scenarios in the same manner as with the real environment (Rothbaum et al., 2006). The intensity of the emotional content of the simulation is controlled by a clinician and adjusted to the individual needs of a patient (Rizzo et al., 2007). VRET provides advantages over traditional ET where time and environment are restricted and difficult to control, or that may potentially expose a patient to danger if delivered *in vivo*. Moreover, it offers a tool that could facilitate exposure for avoidant patients, as well as emotional engagement (Rothbaum and Hodges, 1999). Furthermore, VRET allows for better experimental methodology and design as it improves protocol standardization, and control over duration and type of stimuli delivered (Rizzo et al., 2013).

VRET efficacy has been demonstrated in four meta-analyses (Parsons and Rizzo, 2008; Powers and Emmelkamp, 2008; Opriş et al., 2012), with evidence that the treatment effects transfer to the real world (Morina et al., 2015). The new generation of VR systems are becoming more affordable and accessible for customers at the clinics and patients homes, beginning a technological revolution in mental health, allowing a treatment in more ecologically valid conditions (Slater and Sanchez-Vives, 2016). Psychotherapy experts forecast that VR therapy would be one of the major approaches in the future of the mental health care (Norcross et al., 2013). Furthermore, the study conducted by Garcia-Palacios et al. (2001) demonstrated that 80% of participants with a specific phobia, would choose VRET over traditional ET.

Mechanisms underlying ET and VRET are often explained in terms of fear inhibition and inhibitory learning (Wilhelm et al., 2005; Craske et al., 2012). Inhibition is characterized as the suppression of irrelevant and unwanted response, stimulus, memory or emotion (Aron, 2007). The inhibitory function allows for a transition to a response which is more relevant to goal or circumstances (Barkley, 1997). Deficits in inhibitory regulation have been associated with many psychiatric disorders including substance abuse, antisocial and borderline personality disorders, bipolar disorder, anxiety, phobias, and PTSD (Moeller et al., 2001; Jovanovic et al., 2009). Inhibition of fear responses is the ability to discriminate between danger and safety signals and suppress fear responses in the presence of safety cues (Jovanovic et al., 2012). Inhibitory learning derives from habituation and extinction learning, and involves confronting fear-eliciting stimuli in the absence of negative results, and learning new no-threat associations between neutral and feared stimuli until the level of anxiety diminishes (Craske et al., 2008).

The neural mechanisms underpinning traditional ET have been widely researched. In neuroscience terms, ET attempts to restore a balance within a fear circuit involving the prefrontal cortex (PFC) and amygdala (McNally, 2007). PFC mediates fear inhibitory response and emotional reprisal in ET (Quirk et al., 2006; Jovanovic and Norrholm, 2011; Craske et al., 2012). Specifically neurons in the medial prefrontal cortex (MPFC) may have direct inhibitory action on the amygdala, and in the dorsolateral PFC (DLPFC) have indirect inhibitory function over amygdala, which exhibits exaggerated activity during exposure to fear-evoking stimuli in clinical population (Phelps et al., 2004; Hartley and Phelps, 2010). It has been suggested that the neural mechanisms of fear inhibition overlap with mechanisms of cognitive emotional regulation and fear extinction (Hartley and Phelps, 2010). In particular, DLPFC and MPFC which inhibit anxiety-related activity in the amygdala, mediate a process of reappraisal of negative emotional stimuli and emotional regulation (Ochsner et al., 2002; Ochsner and Gross, 2005; Wager et al., 2008). Therefore activity in DLPFC might reflect a therapeutic strategy for anxiety disorders which aim to facilitate cognitive control of fear through a reappraisal of negative emotional stimuli (Ochsner et al., 2002). Previous neuroimaging studies performed on healthy participants found increased activity in the MPFC and DLPFC during perception of fearful pictures (Lange et al., 2003), fearful faces (Nomura et al., 2004) or suppressing negative mood during decision making (Quirk and Beer, 2006). Moreover, studies found that increased activity in DLPFC and MPFC has an inverse relationship with amygdala activity (Quirk et al., 2006). In contrast, studies comparing healthy controls to patients with phobias, anxiety and PTSD, demonstrated decreased activation in DLPFC and MPFC in the patient population (Etkin and Wager, 2007). This was correlated with increased activity in the amygdala when exposed to fear-evoking stimuli (Shin and Liberzon, 2009). Hypoactivation in MPFC and DLPFC could indicate deficits in fear inhibition and reappraisal, while hyperactivation in the amygdala could indicate an abnormally exaggerated response to threat (Duval et al., 2015).

Despite evidence showing how ET changes the fear circuit after the treatment, little is known about what is happening within the session (Åhs et al., 2017). Employing within-session brain imaging could help therapists objectively monitor patient’s neural response in real time, and determine the optimal level of exposure for the treatment effect to occur (Brouwer et al., 2011). Within-session treatment effects have been observed during ET session in patients with specific phobias, as measured by self-reports (Zlomke and Davis, 2008). However, there has been little research on the neural basis of within-session changes during ET. Two previous brain imaging studies investigated the effects of single ET session on the brain by measuring within-session changes in neural activity in phobic participants during repeated exposure to fearful stimuli. The first study conducted by Veltman et al. (2004) compared brain activity from acrophobic participants to healthy controls employing a symptom provocation paradigm. The result showed neural habituation effects in the bilateral anterior medial temporal lobe, including the amygdala, represented in decline of Regional Cerebral Blood Flow (rCBF) in arachnophobic participants during repeated exposure to pictures of spiders. Particularly, the right amygdala activity demonstrated habituation effects between 5 min and 15 min of exposure. More recently a study conducted on participants with social anxiety disorder found within-session reduction in amygdala rCBF, correlated with decreased subjective anxiety ratings and drop in heart rate during the stressful speech in front of an audience—from one speech (block) to another (after 2.5 min; Åhs et al., 2017).

Although the aforementioned studies have provided some insight into the neural activity changes during ET sessions, little is known about how VRET affects neuronal response, moreover so far there are no studies which have investigated within-session brain activity during VRET.

Combining VRET with neuroimaging aids the tailoring of more efficient interventions, evaluation of treatment effects, confirmation of their ecological validity and potential benefits or directions in research and clinical application (Chou et al., 2012). The neural basis of inhibitory response in VRET, and its treatment effect on the brain function have been investigated using functional magnetic resonance imaging (fMRI) combined with VR in Cue ET for nicotine cravings (Lee et al., 2005; Moon and Lee, 2009), driving with distractions (Schweizer et al., 2013), fear conditioning (Alvarez et al., 2008, 2011) and animal phobia (Clemente et al., 2014). Lee et al. (2009) employed electroencephalographic (EEG) in VR to measure brain activity during exposure to alcohol cues in patients with alcohol dependence. One study combined wireless EEG with desktop VR assessing brain activity emotional regulation in mood inducing simulation (Rodríguez et al., 2013, 2015). However, to the best of our knowledge, the study conducted by Roy et al. (2010) is the only randomized controlled trial study that employed brain imaging techniques to investigate VRET for PTSD. This used fMRI before and after the treatment to assess improvement in brain function after VRET. The result of the study revealed decreased activity in the amygdala, anterior cingulate cortex and increased activity in the lateral PFC. In line with previous neuroimaging studies on ET, research on the neural mechanisms underpinning inhibitory response in VRET demonstrated a role of the PFC, in particular, the DLPFC and MPFC.

However, we argue that the previous studies employed technologies that do not effectively balance quality of measurement and naturalness of response. First, previous studies employed fMRI or EEG. Restraint within a large noisy fMRI scanner not only restricts freedom of movement, but could also evoke anxiety, especially in patients with anxiety-related disorders (Irani et al., 2007). Recently compact, wireless and portable EEG has been combined with VR displays in which people can more freely move (Török et al., 2014). However, the disadvantages of EEG include susceptibility to motion artifacts, electronic signal interference (Islam et al., 2016), and low spatial resolution (Fazli et al., 2012). On the other hand, functional near-infrared spectroscopy (fNIRS) offers the potential to bridge the gap between fMRI and EEG within VR. Specifically, it is anticipated that it will allow more natural movement while providing intermediate spatial resolution and less susceptibility to motion artifacts and electrical noise (Piper et al., 2014). Holper et al. (2010) were the first to combine semi-immersive desktop VR with fNIRS as a tool for monitoring virtual rehabilitative training. Other recent studies showed fNIRS can be used in combination with desktop VR in balance control (Moro et al., 2014), or navigation learning (Ayaz et al., 2011). Head-Mounted Display (HMD) based VR was combined with fNIRS for the first time in the virtual version of line bisection task (Seraglia et al., 2011). Furthermore, a method of immersion in VR impacts on the naturalness of experience (Sander et al., 2006; Diemer et al., 2015). HMDs are commonly used in research or therapy (Simone et al., 2006). One of the disadvantages of HMD is that it hides others or a therapist from the view of the user (Roberts et al., 2016). Moreover, it also restricts natural locomotion, tethering the user to the computer, in addition hiding real-world hazards (Juan and Pérez, 2009). On the other hand, CAVE-like systems (Cruz-Neira et al., 1993) have potential to address many of those issues, immersing a user into a surrounding room-sized VR simulation that supports both natural locomotion and interaction with a simulation, in the space without losing the sight of one’s own body or others (Muhanna, 2015). CAVE-like systems could be particularly useful as a tool for delivering VRET as they allow natural movement within the simulation. Coelho et al. (2008) emphasized a role of movement in acrophobia treatment. The study showed that anxiety levels were higher in patients who were physically moving during exposure to heights, and the locomotion also improves a sense of presence (Slater et al., 1998). Such Cave–like systems provide an opportunity for combining with portable brain imaging devices without obscuring data quality due to the movement or sensor displacement when using HMDs (Landowska et al., 2018).

The current study has both neuroscientific and methodological contributions. This study contributes to the understanding of the neural basis of VRET by measuring both within-session and between-session fear inhibition and reappraisal during an ET by employing fNIRS. Additionally, it contributes to methodology in both neuroscience research and ET by using a combination of neural imaging and VR technology that not only promotes ecological validity but would also allow a participant or client to fully share the experience with a mental health professional.

Although neural mechanisms of ET are already known, there has been little study of within-session effects during ET, or impact of VRET on functional brain activity. Moreover, there are no studies that have looked at both at the same time. Although VRET has been combined with neuroimaging before, we argue that previous studies have used technologies that do not adequately balance quality of measurement and naturalness of response. Specifically, they employed technologies which limit participant’s locomotion and obstruct the view of one’s body. Therefore, to promote ecological validity, this study employed wireless brain imaging and a large CAVE-like Immersive Projection Technology (IPT). Combining wireless fNIRS with IPT systems allows more natural movement without losing the sight of one’s own body while maintaining a data quality.

The aim was to measure brain activity within the PFC, both within and across VRET sessions, using a combination of technologies that provide reasonable resolution while promoting ecological validity and fit to clinical use. Fourteen volunteers (*N* = 14) with mild acrophobia, assessed with Height Anxiety Questionnaire (HAQ; Cohen, 1977), took a part in three-session VRET involving two virtual rooms, in which one appeared to have much of the floor missing, revealing a (virtual) drop below. No other therapy was given. Neural activity was measured both within—and between sessions. For within-session changes in oxygenated (HbO) and deoxygenated (HbR) hemoglobin concentration changes in the PFC were measured in three blocks using fNIRS. For between sessions we investigated the difference in HbO and HbR in the PFC between the first block of the first day and the last block of the last day of exposure. A key methodological objective was to maximize ecological validity by allowing freedom of movement and sight of one’s own body. This was approached by combining wearable fNIRS within IPT. The stimuli were adapted from a classic VR presence experiment—Pit room (Meehan et al., 2002), which demonstrated a psychophysiological response to virtual heights.

The study hypothesized that:

1. Participants with moderate acrophobia exposed to virtual heights will fail to activate PFC when exposed to virtual heights at the beginning of VRET, as measured by HbO and HbR
2. The activity in the PFC will increase over the time from block to block
3. The activity in the PFC will increase over the time from session to session
4. Subjective Units of Distress (SUDS) will drop over time from block to block
5. SUDS will drop over time from session to session
6. Activity in the PFC would be negatively correlated with SUD scores
7. Activity in the PFC would be correlated with the initial subjective HAQ scores

## Materials and Methods

### Participants

Fourteen (*n* = 14) participants (12 females, two males, mean age *M* = 42.30, *SD* = 16.57) with a moderate fear of heights were recruited from the Anxiety UK charity (4) and staff and student communities of the Universities of Salford (1) and Manchester (9). Each participant was pre-screened for the fear of heights using HAQ (Cohen, 1977). Only participants with a medium of fear of heights, who scored higher than one-third of the questionnaire scores, but lower than three-thirds (mean score above 41 = 30% and below 90) were invited to participate (Steinman and Teachman, 2011). Participants were also excluded from this study if they had suffered an epileptic episode, have felt unwell during a VR or 3D cinema experience, often suffer from a migraine, or have skin that is excessively sensitive and thus might get damaged by sensors. All participants were presented with the Participant Information Sheet (PIS), which advised them of the potential risks associated with the experiment such as cybersickness and discomfort related to the brain monitoring module. The PIS also informed participants about data anonymization and confidentiality and they were advised that they could withdraw from the experiment at any time without giving reasons. Ethical approval HSCR 15/88 was granted by the University of Salford’s Health Science Ethics Committee. All procedures performed in studies involving human participants were in accordance with the ethical standards of the institutional and/or national research committee and with the 1964 Helsinki Declaration and its later amendments or comparable ethical standards. Written Informed consent was obtained from all individual participants included in this study.

### Instruments

#### Immersive Projection Technology (IPT) Octave

**Figure 1:**
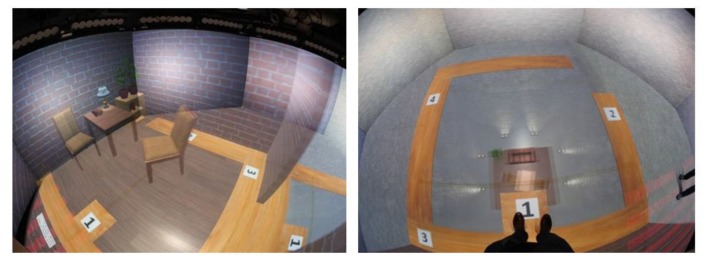
Experimental stimuli: left—view in the training room, right—view looking down into the room below.

Octave is an octagonal cave-like IPT space (Figure 1). It is larger than most IPT’s including CAVEs and is approximately 5 m across. This is big enough to allow: the patient to move away from or toward a threat; natural walking throughout the task and a therapist to join the client in clinical treatment. Parallax and stereo work together to give the feeling of moving within the room and to give the feeling of depth to the drop. The participant sees seemingly holographic images when wearing stereo glasses. The glasses were XPAND 3D Shutter Glasses Lite RF (X105-RF-X1). The eyes are alternately shuttered in synchronization with respectively offset views of the simulation through the active stereo. The shuttering frequency 96 of Hz which means that stereo is delivered at 48 Hz. The alternating images are delivered via the surrounding walls and floor display. There are eight surrounding wall screens 2,600 mm × 1,969 mm with resolution 1,400 × 1,050 pixels, and 96 Hz refresh rate. There are 14 Christie S + 3K mirage projection units of which six cover the floor and eight rear wall projectors. Octave image is generated by the workstation with 2× Xeon E5-2650 giving 32 threads, 64 GB memory, SSD and 4× Nvidia K5000 with the k-sync card running a single desktop through 4 mosaic instances. Parallax is provided by updating viewpoint from an optical motion tracking system. The Vicon MX-F40 tracking uses custom designed optical markers on the glasses and is controlled by Dell workstation running Windows 7 and Vicon Tracker 2.0. This tracks the position and orientation of the user’s head so that the system refreshes the displays according to head orientation and position, allowing for the creation of head- movement parallax. The immersive acoustic system is controlled by Mac Pro with 2 × Intel^®^ Xeon^®^ CPU X5570 @ 2.93 GHz and 3.06 GHz, 32 Gb RAM running Windows 7 (64-bit).

#### Functional Near Infrared Spectroscopy (fNIRS)

In order to investigate the neural basis of inhibitory response and function we utilized fNIRS, which measures changes in a hemodynamic response associated with the neural activity from the sub-surface of the brain using near-infrared light (Hoshi and Tamura, 1993; Kato et al., 1993; Villringer et al., 1993).

**Figure 2:**
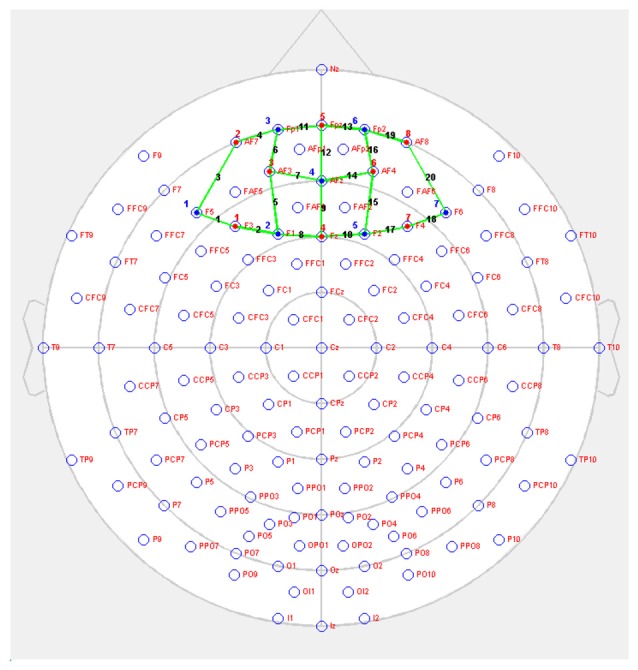
NIRSport probe setup on prefrontal cortex (PFC; red = sources, blue = detectors, green = channels).

Changes in brain oxygenation concentrations were measured using NIRSport (NIRsPORT 8-8, NIRx Medizintechnik GmbH, Berlin, Germany). This is a portable, wearable, battery-operated multichannel fNIRS system consisting of eight LED illumination sources and eight active detection sensors, which can be arranged in 64 channels^1^. Twenty channels were set up covering PFC. Emitters were placed on positions F3, AF7, AF3, Fz, Fpz, AF4, F4, AF8, while detectors were placed on positions F5, F1, Fp1, AFz, F2, Fp2 and F6. The source-detector distance was 3 cm (Figure 2). Optodes were placed on the participant’s head using EasyCap^2^ relative to the international 10/20 system.

### Stimuli

The environment consisted of two rooms: the training room and the pit room. The scenario was created using Unity 5 game engine (academic version 5.1.0f3 64-bit^3^). To run the simulation in Octave MiddleVR for Unity (version 1.6.1f6 was used^4^) was used. MiddleVR is a middleware solution that allows for a connection of VR peripheral devices such as tracking, projectors and controllers. Both rooms had the virtual wooden plank 40 cm wide on the inside of the walls, on which the participant was asked to walk. The training room looked like a normal room with floor and furniture and the virtual plank was placed directly on the floor. The pit room had no floor but the virtual wooden plank and the room below. The room, approximately 6 m below the plank, was decorated and had furniture (Figure 2).

### Procedure

Each participant was provided with the PIS and HAQ at least 24 h prior to the experiment. The HAQ, developed by Cohen (1977) is a 20-item self-report measure which assesses the severity of anxiety related to heights. Participants rated their anxiety on the 7-point scale ranging from 0 (not anxious at all) to 7 (extremely anxious) and returned to the researcher to determine eligibility. On arrival they were instructed about safety in Octave and given a consent form to sign if they agreed to do the experiment. Then they were introduced into the Octave and the simulation. The researcher explained the task and instructed participants about the level of the movement they were allowed to perform in order to minimize the motion artifacts in the data. Then participants were allowed to familiarize themselves with the virtual environment for about 5 min. Following practice and familiarization participants were fitted with the NIRSport. The fNIRS system was then calibrated for the optimal amplitude and signal-to-noise ratio (SNR). The quality of the optical densities was then assessed visually by the researcher. If the level of the noise in a data was too high, the researcher readjusted any noisy optode ensuring optimal contact with a scalp. The retaining cap, which reduces the ambient light and reduces the risk of optode displacement, was placed over the EasyCap. The data was recorded using a battery-operated fNIRS system which is powered by the laptop and the data is saved and stored on the hard drive. The fNIRS battery and the laptop were placed in the backpack which must be worn by a participant during the whole experimental session. After the preparation participants were led to Octave and asked to perform a simple walking task on the wooden plank in both training and pit room. Participants were asked to rate their SUDS of fear on a Likert scale ranging from 0 to 100, where 0 indicated “not at all anxious,” and 100 indicated “extremely anxious” (Wolpe, 1973). The researcher recorded the SUDS scores during each break between blocks. The procedure for each of three sessions was the same, except that the familiarization phase was conducted only on the first day. After the final VRET session, participants were asked to fill in two questionnaires—Cybersickness and IPGroup Presence Questionnaire (IPQ) to evaluate user’s general experience related to the quality of the VR system and simulation. IPQ is the 14-item scale used for measuring subjective sense of presence in VR. Participants are asked to rate their presence on a 7-point Likert scale. The three subscales which assess different components of presence: spatial presence (consisting of five items), involvement (consisting of four items) and realism (consisting of four items). Moreover, there is one additional item to assess general presence (Schubert et al., 2001). Simulator Sickness Questionnaire (SSQ)—is the 16- item tool that assesses possible side effects of VR exposure on a 4-point Likert scale (Kennedy and Fowlkes, 1992).

### Experimental Design

**Figure 3:**
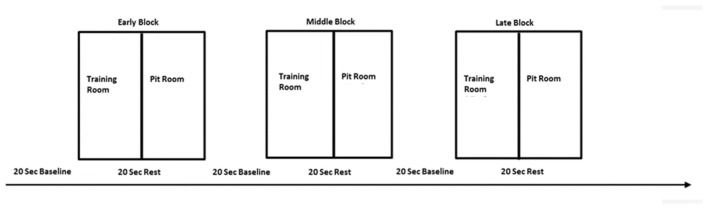
The experimental design—one session consisted of three blocks—early, middle and late, separated with 20 s baseline. Each block consisted of two conditions—training room and pit room separated with 20 s rest. The same procedure was repeated over three virtual reality exposure therapy (VRET) sessions.

This experiment employed within-subject design in order to reduce the number of errors in the variance due to natural human variances in the data and to test a difference between conditions. All participants were tested under the same conditions for each of three sessions. For the task, we adopted blocked design. The whole experimental session consisted of three blocks: early, middle and late. This approach was adopted to investigate brain activity related to within-session learning. Each block lasted 240 s and was proceeding with the pre-task 20 s baseline (Figure 3). The blocked design was employed in order to facilitate naturalness of the response and mimic the real-life situation in which usually there are no inter-stimulus periods or breaks.

There were two experimental conditions within each block, each lasting 120 s: training room and pit room. Each condition was preceded with 20 s rest for the hemodynamic response to return to the baseline. Block sequences between subjects were randomized—participants were instructed to move to either training room or pit room. During the session participants heard pre-recorded audio instructions: “Please take a rest,” “Move to the training room” or “Move to the pit room.” The experiment started with 20 s baseline prior the first stimuli onset, when participants were instructed to step outside the simulation. They were instructed to stay still, close their eyes, clear their mind and relax. After the baseline participants heard audio instruction to move either to the training room or move to the pit room in random order. The task in both rooms was the same—participants were walking on the plank in the training room for 120 s and walking on the plank in the pit room for 120 s. There were 20 s breaks between each condition and each of the blocks in order for the hemodynamic response to return to the baseline. During the between-blocks break, participants were asked to rate their discomfort on the SUDS scale. The experimenter was present in the experimental area during the whole experiment placing the experimental markers on the data manually using keyboard every 20 s. The presence of the researcher also provided a safety cue for the participant to minimize a risk of anxiety when exposed to virtual height. The data was transferred over the network using HotKeyNet (^5^). The same procedure was repeated for all of three sessions. After each session participants were asked to return for the next session next day or maximum within 48 h. This approach was taken in order to minimize the risk of participants being exposed to heights in real life between sessions. As we wanted to investigate only the effect of virtual heights on acrophobia, participants were instructed to avoid any situations involving heights in daily life between sessions, until the experiment was completed. After the last sessions participants were asked to fill in two questionnaires: IPGroup Presence; and Cybersickness (Kennedy and Fowlkes, 1992). Afterwards, there was a short informal interview with the researcher in order to gather the feedback about the experiment and ensure that the participant experienced no side effects and could safely leave the lab.

### Data Acquisition

The data was acquired with the NIRStar acquisition Software (version 2014, NIRx Medical Technologies LLC) running on Windows 7 (64-bit) laptop with Intel^®^ Core™ i5-4200 CPU @ 1.60 GHz 2.30 GHz with 4.00 GB RAM. The blood oxygenation was measured at two near infra-red light wavelengths of 760 nm and 850 nm, with the sampling rate 7.81 Hz.

### Data Analysis

Preprocessing and statistical data analysis was performed using Statistical Parametric Mapping NIRS-SPM (SPM 8; Friston, 2007; Ye et al., 2009) analysis tool implemented in NIRSLab (version 2017.6) to identify brain regions activated during the virtual height exposure. Data was first inspected in order to identify noisy channels. Due to motion artifacts two participants were excluded from the data analysis, therefore 12 participants were included further in the analysis. The coefficient of variation (CV) was used in order to quantify the signal-to-noise for the raw time series for each participant and each channel. Channels which showed CV with the value exceeding 15% were rejected from the analysis (Piper et al., 2014). Raw data was converted to the hemoglobin concentration changes using the modified Beer-Lambert Law (Delpy et al., 1988) for or each channel, each block, each day and each subject. Oxy-(HbO), deoxy-(HbR) and total-(HbT) hemoglobin time series were band-pass filtered with low cut-off frequency of 0.0.0083 Hz and high cut-off frequency of 0.2 Hz, to remove drifts and respiration, and cardiac effects from data (Piper et al., 2014; Naseer and Hong, 2015). A differential path length factor of 7.25 for 760 nm and 6.38 for 850 nm was applied (Essenpreis et al., 1993). Molar extinction coefficients ε for HbO at 760 nm = 1486.5865 cm^−1^/M and 850 nm = 2526.391 cm^−1^/M, were applied from W. B. Gratzer, Med. Res. Council Labs, Holly Hill, London and N. Kollias, Wellman Laboratories, Harvard Medical School, Boston, MA, USA. Data were modeled with GLM. The regressors were modeled via convolution by the 120-s box car function provided by SPM8. Discrete cosine transform basis function was used for temporal filtering and precoloring HRF was used for the serial correlations (Ye et al., 2009). In the first level channel-wise analysis t-contrasts were then created for HbO and HbR concentration changes, to generate statistical parametric maps of activation for two regressors: training and pit, for each channel and each participant. The data for both conditions (training and pit) was baseline corrected. A separate baseline was recorded prior to each block. SPM t-maps were generated by using two contrasts: training-pit and pit-training, and thresholded at *p* < 0.05 (corrected). At the group analysis SPM HbO and HbR t–statistics were calculated to identify the channel significantly activated by exposure to virtual heights with the significance level set at *p* < 0.05 (corrected). The estimated anatomical location of each channel was determined using anatomical locations of international 10-10 system cortical projections of EEG sensors (Koessler et al., 2009).

Neuronal changes associated with between-session effects were determined by calculating the differences between the last block of the last session and the first block of the first session. First beta coefficients were retrieved from the first level analysis for each subject, channel, block and session. Then results from the first session were extracted from results from the last session using MATLAB (R2017b), resulting in sets of paired betas for each subject and channel. The paired-beta images were further analyzed using group-wise contrast analysis in NIRSLab SPM. The significance level was set up at *p* < 0.05 (corrected).

In order to investigate the effect of within-session changes in brain activity, HbO and HbR beta values were extracted from the first level analysis for each participant, session, block and channel. These were averaged across channels and blocks and further analyzed in SPSS, due to the limitations of NIRS software.

The Region of Interest (ROI) analysis provided a third level analysis. ROIs were defined* a priori* across all the channels, using the probabilistic assessment of cortical projection sensors, underlying 10-20 system anatomical surface locations (Koessler et al., 2009). Four ROIs were defined on the basis of BA atlas (Brodmann, 1909) to be represented by channels: 1, 3 and 7 (Left DLPFC); 9, 14, 18 and 20 (Right DLPFC); 4, 6 and 11 (Left MPFC); and 12, 13, 16 and 19 (Right MPFC) respectively. The HbO and HbR beta-estimates from those channels were extracted for each subject from the first level analysis and then averaged within each of the ROIs across the selected channels for each session and block. Average beta estimates were then analyzed with SPSS. The within-subject ANOVA was used to test for a difference between blocks. Mauchly’s test of sphericity was performed to assess for the assumption of sphericity. When assumption of sphericity was violated, the Greenhouse-Geisser adjustment was used. A Pearson’s and Spearman’s correlation coefficient was calculated to assess the relationship between HbO and HbR ROIs, and questionnaire data.

Preparation and analysis of the questionnaire data were conducted in SPSS (IBM SPSS, Version 24). All the data were checked for normality using SPSS function “Explore” and Shapiro-Wilk test. The SUDS data, which deviated from normal distribution was analyzed using non-parametric Friedman and Spearman’s correlation tests. Descriptive statistics (means, medians and standard deviations) were calculated for all the questionnaires.

## Results

### Within-Session Learning

#### fNIRS Data

**Figure 4:**
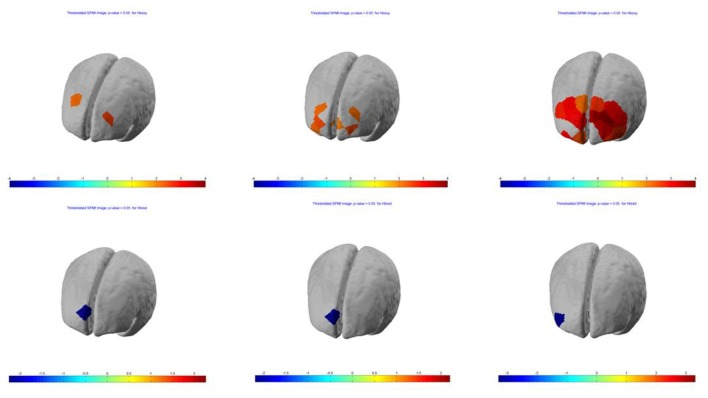
Session 3—group (*n* = 13) Statistical Parametric Mapping (SPM) activation t-maps of oxygenated (HbO; top) and deoxygenated (HbR; bottom) pit room > training room, threshold level *p* < 0.05 for each of three blocks 1-early, 2-mid and 3-late, respectively. The color bar represents *t*-values.

SPM contrast analysis for the group effects (pit room > training room), at the significance threshold level *p* < 0.05 (corrected), revealed no significant difference between training and pit conditions during both first and second session of the experiment. Moreover, for both first and second session there was no significant effect of the blocks. However, the group demonstrated significant results during the third session. SPM contrast analysis (pit room > training Room) at the significance threshold level *p* < 0.05 (corrected), revealed a significant increase of HbO concentration changes in the bilateral DLPFC during first block, and in the bilateral DLPFC and bilateral MPFC during the second and third block. Additionally, a significant HbR decrease was observed in the right DLPFC during all three blocks. All the results from the third are summarized in Table 1 (*t*-values, Broadman areas and anatomical labels) and Figure 4 (SPM t-maps).

**Table 1:** Summary of results for all three blocks for oxygenated (HbO; channel number, *t*-value, anatomical label and Brodmann areas).

Block	Channel	*T*-value	Label	Brodmann area
1	Channel 5	2.57	DLPFC L/ Frontal Eye Fields	BA 8/9
	Channel 17	2.16	DLPFC R/ Frontal Eye Fields	BA 8/9
2	Channel 5	2.20	DLPFC L/ Frontal Eye Fields	BA 8/9
	Channel 6	2.43	MPFC L	BA 10
	Channel 12	2.09	DLPFC B	BA 9
	Channel 15	2.23	DLPFC R/ Frontal Eye Fields	BA 8/9
	Channel 16	2.38	MPFC R	BA 10
	Channel 19	2.17	DLPFC R	BA 46
3	Channel 1	3.06	DLPFC L	BA 46
	Channel 2	3.15	Frontal Eye Fields	BA 8
	Channel 3	2.70	DLPFC L	BA 46
	Channel 4	2.68	DLPFC L	BA 46
	Channel 5	3.80	DLPFC L /Frontal Eye Fields	BA 8/9
	Channel 6	3.15	MPFC L	BA 10
	Channel 7	3.28	DLPFC L	BA 9
	Channel 8	2.72	Frontal Eye Fields L	BA 8
	Channel 9	3.70	DLPFC R	BA 9
	Channel 10	2.22	Frontal Eye Fields R	BA 8
	Channel 13	2.45	MPFC R	BA 10
	Channel 14	3.17	DLPFC R	BA 9
	Channel 15	3.11	DLPFC R /Frontal Eye Fields	BA 8/9
	Channel 17	3.05	Frontal Eye Fields R	BA 8
	Channel 18	2.77	DLPFC R	BA 46
	Channel 19	3.11	DLPFC R	BA 46

*All p values < 0.05 (corrected)*.

To investigate difference between blocks during a third session, we further performed a 3 × 2 repeated measures ANOVA with two within-subject factors—block (early, mid and late) and condition (training and pit) for HbO and HbR. The results revealed a significant HbO effect of the block *F*_(2,24)_ = 4.970, *p* = 0.016, and a significant effect of the condition *F*_(1,12)_ = 23.416, *p* = 0.001. The interaction between block and condition was not significant *F*_(2,24)_ = 1.1437, *p* = 0.259. *Post hoc* comparison using Bonferroni correction indicated that there was a significant increase in HbO between block 1 and block 3 (*p* = 0.038, mean difference in betas *M* = 0.000444), and HbO concentration changes were significantly higher in the pit room (mean difference in beta values = 0.000517, *p* = 0.0.01) regardless the block. The HbR analysis showed no significant effect of block *F*_(2,24)_ = 1.305, *p* = 0.290, however there was a significant effect of condition between pit and training *F*_(1,12)_ = 17.165, *p* = 0.001 (mean difference in betas *M* = 0.000113).

#### SUDS

Means, medians and SDs for SUDS during VRET are presented in Table 2. The Friedman test demonstrated that there was a significant difference in SUDS depending on the block and across all three sessions of VRET $\chi_{\left(8\right)}^{2}$ = 62.387, *p* = 0.001. *Post hoc* analysis was conducted using series of Wilcoxon signed rank tests.

**Table 2:** Subjective units of distress (SUDS) for each session and each block—means, standard deviations and medians.

SUDS	*M*	*SD*	Median
Day1_Block1	53.0769	18.87883	50.0000
Day1_Block2	50.0000	19.03943	50.0000
Day1_Block3	43.8462	15.56624	45.0000
Day2_Block1	43.4615	14.19868	40.0000
Day2_Block2	38.0769	14.22124	40.0000
Day2_Block3	34.2308	13.66964	30.0000
Day3_Block1	32.3077	15.76063	30.0000
Day3_Block2	25.7692	14.83888	20.0000
Day3_Block3	20.3846	12.98421	20.0000

Session 1—The results showed that SUD scores dropped significantly between first and second block (*Z* = −1.994, *p* = 0.046) and second and third block (*Z* = −2.588, *p* = 0.010).

Session 2—The results showed that SUD scores dropped significantly between first and second block (*Z* = −2.739, *p* = 0.006) and second and third block (*Z* = −2.428, *p* = 0.015).

Session 3—The results showed that SUD scores dropped significantly between first and second block (*Z* = −2.598, *p* = 0.009) and second and third block (*Z* = −2.622, *p* = 0.009).

### Between Session Learning

#### fNIRS

**Figure 5:**
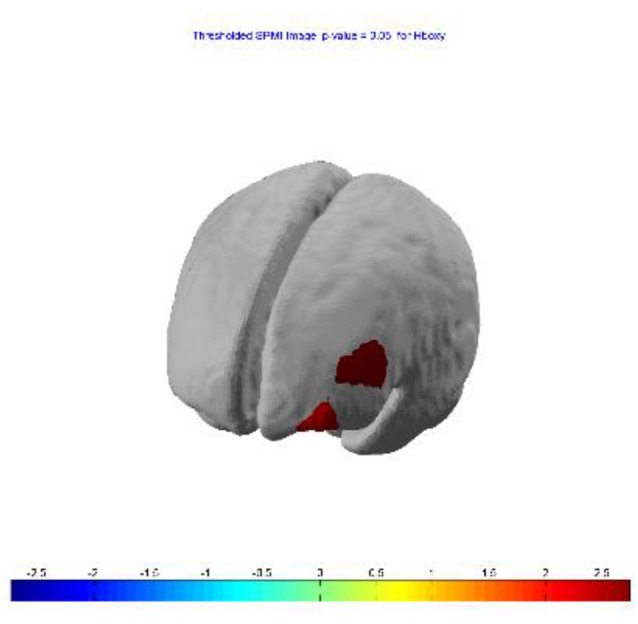
Between-session (last lesion − first session) t-contrast, revealed significant increased HbO in left dorsolateral PFC (DLPFC) during exposure to virtual heights.

SPM contrast analysis (last session > first session; pit room > training room) at the significance threshold level *p* < 0.05 (corrected), revealed a significant increase of HbO concentration in the left DLPFC (Figure 5) in channel 1 (*t*-value 2.74) and channel 4 (*t*-value 2.28). The HbR analysis showed no significant results for between-session effects.

#### SUDS

There was no significant drop in SUDS from the first to the second session (*Z* = −0.240, *p* = 0.810), however, there was a significant difference in SUDS between second and third session (*Z* = −2.684, *p* = 0.007). Means, medians and SDs for SUDS during VRET are presented in Table 2.

### Correlations

**Figure 6:**
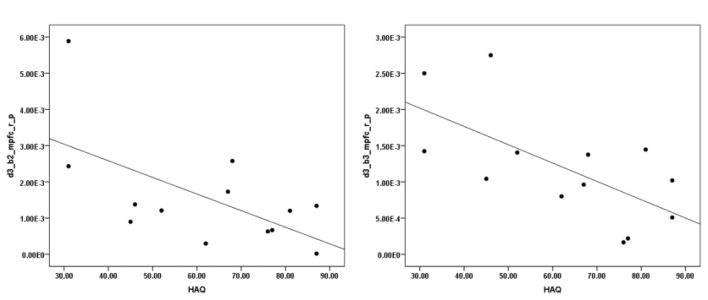
Correlation between initial severity of acrophobia (height anxiety questionnaire (HAQ)) and HbO changes in the pit room during the second (left) and third (right) block.

The correlation analysis between HAQ and each of the ROIs revealed a significant negative correlation between HbO in the R MFPC and HAQ during exposure to virtual heights in the middle block (*r* = −0.603, *N* = 13, *p* = 0.029, two-tailed) and late block (*r* = −0.650, *N* = 13, *p* = 0.016, two-tailed; Figure 6). There was no significant correlation between SUDS and presence scores and any of the ROIs.

### Questionnaires Analysis Results

The IPQ was used in order to assess the quality of the simulation. In general participants reported moderate level of presence (overall mean presence score was 4.43, SD 1.02) which indicates that employing fNIRS in VR did not significantly impact on user’s experience. Results are presented in Table 3.

**Table 3:** Presence scores measured by the IPG—means and standard deviations.

Presence	Mean	Standard deviations
Spatial presence	5.29	0.93
Involvement	3.96	1.27
Realness	3.96	1.28
General presence	5.38	0.96
Total average presence	4.65	0.95

The correlation analysis between each of the ROIs and presence revealed a significant positive correlation between HbO in the DLPFC R during exposure to virtual heights in the late block of the last session and presence (realness; *r* = 0.563, *N* = 13, *p* = 0.045, two-tailed).

Cybersickness questionnaire was used in order to assess user’s experience and safety related to the VR system. Most of our participants scored reasonably low on the questionnaire (mean score 3.23, SD = 3.00), only two of them experience slight symptoms of Cybersickness. The result demonstrated that the Octave system combined with fNIRS may cause only a slight cybersickness in some participants, but generally is safe for phobic participants.

## Discussion

The aim of this study was to measure brain activity in the PFC, both within and between VRET sessions in participants with moderate acrophobia. Additionally, this study investigated the correlation between brain activity and subjective fear ratings (SUDS and HAQ) within and between VRET sessions. A key methodological objective was to maximize ecological validity by allowing freedom of movement and sight of one’s own body. This was approached by combining wearable fNIRS with large IPT. The stimuli were adapted from a classic VR presence experiment—Pit Room (Meehan et al., 2002), which demonstrated a psychophysiological response to virtual heights in healthy participants.

The analysis showed no difference in brain activity between the training room (control condition) and the pit room (virtual heights condition) at the beginning of VRET, that indicates that participants with acrophobia fail to activate the DLPFC and MPFC when exposed to fear-evoking virtual stimuli. The result is consistent with previous neuroimaging studies which suggested that participants with anxiety disorders, phobias and PTSD exhibit functional deficits in activity in the DLPFC (Etkin and Wager, 2007; Straube et al., 2007; Hauner et al., 2012; Lipka et al., 2014; Etkin et al., 2015) and MPFC (Quirk et al., 2003; Williams et al., 2006; Liberzon and Sripada, 2007; Shin and Liberzon, 2009). The current study found within-session effects reflected in increased brain activity at the end of VRET in the DLPFC and MPFC. Specifically, at the beginning of the third session (first block), we found increased HbO in the bilateral DLPFC and decreased HbR in the right DLPFC. This pattern of activity then extended to HbO increase in the bilateral MPFC during second block (after 4.2 min) and increased in magnitude during a third block (after 8.4 min). Åhs et al. (2017) found changes in brain activity after 2.5 min of exposure to the fear-evoking pictures, and Veltman et al. (2004) suggested that within-session habituation effect could be detected within 5–15 min. Both of those studies found changes in brain activity in the amygdala, but not PFC, within a single session of exposure. It is possible that changes in the PFC occur more gradually and require more time than neuroplasticity in the amygdala (Takehara et al., 2003). The current study utilized fNIRS which has limited penetration depth (Ferrari and Quaresima, 2012) and therefore cannot measure the signal from the amygdala, however, we demonstrated that within-session neuronal changes in the PFC can be detected in VRET by fNIRS.

This pattern of within-session activation might indicate that VRET initially triggers cognitive reappraisal of virtual stimuli, which next leads to inhibition of irrelevant emotional responses. This could be related to the fact that the DLPFC plays a role in the conscious reappraisal of emotional stimuli and the regulation of emotion (Rauch et al., 2006; Hartley and Phelps, 2010), and the MPFC plays role in emotional inhibition and extinction (Phelps et al., 2004). Although the DLPFC does not have neural connections to the amygdala, it may take advantage of the mechanism of inhibition and extinction learning to reduce fear response via MPFC which projects to the amygdala (Delgado et al., 2008; Hartley and Phelps, 2010). The MPFC has shown to inhibit the amygdala activity in fear inhibition and extinction (Ongür and Price, 2000; Giustino and Maren, 2015). The current study demonstrated that inhibitory learning in VRET occurs during the third session and it is preceded by cognitive reappraisal. This suggests that the VRET-induced brain function normalization does not happen instantly, but rather requires multiple sessions to trigger changes in the brain related psychotherapeutic effect. Although some neuroimaging studies on traditional psychotherapy for phobias showed that even a single treatment session can change brain activity (Hauner et al., 2012), others detected neuronal changes after second session (Schienle et al., 2007), or more sessions (Roffman et al., 2005). On the other hand, fMRI studies on VRET detected changes in brain activity after six sessions in the treatment of nicotine cravings (Moon and Lee, 2009), or 12 or more sessions for treatment of PTSD (Roy et al., 2010). The current study detected changes in brain activity during the third session due to the few potential factors. First, this study recruited participants with a moderate phobia, therefore fewer sessions might be required for an improvement. Second, unlike previous studies, this project utilized IPT VR system combined with wireless fNIRS, which promotes naturalness of movement and response. Using these technologies may facilitate therapeutic effect due to improved ecological validity. Especially movement can be important in the treatment of acrophobia due to its ability to trigger a higher level of anxiety which reflects activation of the fear network, which is necessary for habituation to occur (Coelho et al., 2008). Thirdly, contrary results regarding a number of sessions in VRET could be related to different experimental designs and different durations of the sessions (van Minnen and Foa, 2006). In this experiment, one VRET session lasted only 15 min, which might not be sufficient for the learning to occur and that could be the reason we failed to detect within-session effects in brain activity during first two sessions. However, studies on perceptual learning suggested that the learning of new skill requires a period of consolidation (Hauptmann et al., 2005), therefore inhibitory learning might not occur during early sessions of VRET. Further studies should investigate what is the optimal duration of the efficient VRET treatment during a single exposure. Another potential factor influencing a lack of within-session effects at the beginning of VRET could be related to the motion artifacts in fNIRS data. Especially during the first session on VRET, we removed noisy channels from data analysis. Most of the volunteers who participated in this study did not have prior VR experience in IPT, therefore increased motion artifact during the first session could be related to orienting response and novelty of the experience (Gogan, 1970). Further studies should take into account the necessary time required for familiarization with VR.

Between-session changes in brain activity were measured as a difference between the first block of the first session and the last block of the last session. The result revealed significant HbO increase in the left DLPFC after VRET, indicating that acrophobic participants were able to reappraise the fear-evoking stimuli after the treatment. The left lateralization of between-session activation could be related to the previous finding which suggested that negative stimuli are processed in the right hemisphere and positive stimuli are processed the left hemisphere (Davidson and Irwin, 1999). Specifically, the left DLPFC supports retrieval of the memory related to positive emotional information (Balconi and Ferrari, 2013). Thus this pattern of activation might reflect down-regulation of fear responses mediating positive reappraisals of threatening virtual stimuli after VRET. HbR did not show significant between-session changes. This could be related to the fact that HbR tends to have lower amplitude and signal-to-noise ratio (SNR) than HbO (Fishburn et al., 2014).

Regarding subjective fear ratings, the results demonstrated a significant drop in SUDS between second and third session, but not first and second, consistent with previous evidence from studies on CBT (Hayes et al., 2008; Norton et al., 2011). Moreover, within-session reduction in subjective fear rating occurred across all three sessions. This indicates that VRET triggers within–session anxiety reduction from the first session, however between–session effects occur from the second session. This might indicate that fear inhibition learning in VRET as measured by subjective anxiety ratings does not occur instantly but requires some time for consolidation. The decrease in SUDS demonstrated that VRET is an effective tool in the reduction of acrophobia symptoms. However, contrary to expectations, we did not find a significant correlation between brain activity and subjective anxiety ratings. Other studies which investigated the neural basis of VRET did not report a correlation between brain activity and subjective reports, therefore it is difficult to compare the results of this study to previous evidence. (Roy et al., 2010; Clemente et al., 2014). Therefore more studies are necessary to establish if the process of neuroplasticity triggered by VRET affects brain regions differently regarding time-scale. On the other hand, this result could be related to the small sample size not being sufficient to detect the correlation. Some studies claimed that subjective or psychophysiological measures often do not correlate with brain activity (Liberzon et al., 1999; Robinson et al., 2013). Studies which investigated brain function during traditional ET found a positive correlation between subjective reports and neural activity in the amygdala and insula (Straube et al., 2006; Schienle et al., 2007), but not in the PFC (Veltman et al., 2004; Åhs et al., 2017). fNIRS does not allow to measure a signal from amygdala or insula (Liu et al., 2015), therefore it is difficult to determine if such a correlation occurs during VRET. Perhaps more fMRI studies are needed to determine the correlation between brain activity and subjective anxiety ratings in VRET.

The current study, however, found a correlation between initial severity of fear of heights and change in brain activity across the final (third) session. These were respectively measured by HAQ and changes in HbO. In particular, we found a negative correlation between HAQ and activity in the right MPFC during the middle and late block. The MPFC is involved in recall and expression of fear extinction memory, that inhibits a fear response (Etkin et al., 2011), after repeated exposure to aversive stimuli (Milad et al., 2006). This might indicate that participants with an initial lower acrophobia might have more ability to learn how to better inhibit fear response when exposed to virtual heights. On the contrary, participants with higher acrophobia score demonstrated lower activity in the MPFC during exposure to virtual heights, which confirms that they might have a less ability to inhibit a fear response during exposure to virtual heights. Perhaps, the number of sessions required before significant improvements is related to the initial severity of the condition. However, investigating this would require a follow-up study.

Combining fNIRS with IPT did not significantly break the quality of a user’s experience. In general, participants reported that they felt present within the simulation. However, in this study presence was not correlated with changes in brain activity during exposure to virtual heights. The evidence of a role of presence in VRET is mixed. While some studies reported a positive correlation between presence and level of anxiety in VRET (Price et al., 2011), other studies failed to demonstrate such a relationship (Krijn et al., 2004). The meta-analysis performed by Ling et al. (2014) found a medium effect size and correlation between self-reported presence and anxiety during VRET. The fact that we did not find a correlation between other presence subscales and brain activity could be related to small sample size. Another factor influencing this result might be related to the lack of prior VR experience of participants, which perhaps require a longer time to familiarize themselves with the experience in order to develop a better sense of presence. Some previous studies suggested that significant correlations between subjective presence and fear ratings are more often found in clinical samples rather than healthy controls in VR studies (Diemer et al., 2016). The current study involved only participants with moderate acrophobia, but not clinical population. Moreover, it is possible that subjective presence ratings do not accurately measure the essential sense of presence, therefore there is a need for more objective—physiological measurements or behavioral observations, to measure presence in VR in future studies (Meehan et al., 2002).

All participants in this study reported relatively low cybersickness, and this appears to demonstrate that combining wireless fNIRS and IPT does not cause negative symptoms and proves this combination of technology to be safe and comfortable for phobic participants.

### Future Directions

Employing brain imaging in VRET could aim not only to understand its neural mechanisms but also could help to identify neural biomarkers as a treatment predictor to determine the intensity and length of VRET, as well as identify potential responders. Employing prediction approaches based on neural biomarkers, and VR as a stimuli delivery medium, have a capacity to ameliorate the accuracy in predetermining a therapeutic response (Ball et al., 2014). Improving mental health diagnosis process became urgent matter recently as there is a growing number of individuals suffering PTSD and anxiety disorders as a result of terrorist attacks, refugee crisis and disasters (Moran et al., 2017). Employing VR combined with brain imaging as a diagnostic tool might further improve identification of traits to govern treatment due to flexibility and controllability of the technology.

### Limitations of the Study

There are several limitations to this study. First, the sample size was limited, though comparable to other similar neuroimaging studies on VRET. The results require replication to increase confidence. Second, we recruited only participants with a moderate acrophobia, therefore future studies should be conducted on the clinical sample to investigate if VRET combined with brain imaging has the same effect on participants with serve phobia, as well as other disorders. Third, all participants received only three sessions of VRET, which might be insufficient to detect lasting changes in the brain. Additionally, the duration of each session was only 15 min. Therefore, the further studies should perhaps involve a bigger sample size, more sessions, and longer sessions. Although this study did not have a control group, it serves as a preparation for a future randomized controlled trial.

## Conclusion

This study demonstrated a significant increase in the PFC activity, indicative of inhibition of fear, during the third session of VRET in phobic subjects. We believe it is the first study to examine within session neural response to VRET. Furthermore, previous studies have demonstrated significant improvements after 8–12 sessions. As the neural basis for ET for phobia are thought to be similar to other disorders, such as PTSD, anxiety and addictions, these findings are likely to be transferable. A further novelty of this experiment was the use of a combination of neural imaging and VR technology that we have argued promotes ecological validity and reduces additional stress for the client. This approach thus lends itself to reproduction, with lower cost display equipment, in clinical and other settings. Such could be incorporated into understanding, diagnosis, resilience training and treatment of a range of anxiety disorders.

## Data Availability

The raw data supporting the conclusions of this manuscript will be available from the corresponding author on reasonable request.

## Author Contributions

AL and DR conceived, planned, designed the experiment and wrote the manuscript with input from all the authors. DR and PE supervised the project. AL carried out the experiment, data acquisition, analysis, processing and interpretation. PE contributed to statistical data analysis, provided critical feedback and proofread the manuscript. AB gave critical advice regarding clinical application and participated in drafting the manuscript and provided a critical review. All authors discussed the results and contributed to the final manuscript.

## Conflict of Interest Statement

The authors declare that the research was conducted in the absence of any commercial or financial relationships that could be construed as a potential conflict of interest.

## Footnotes

^1^http://nirx.net/

^2^https://www.easycap.de/wordpress/

^3^https://unity3d.com/

^4^http://www.middlevr.com/middlevr-for-unity/

^5^http://www.hotkeynet.com/
